# Integrated analysis of *Dendrobium nobile* extract Dendrobin A against pancreatic ductal adenocarcinoma based on network pharmacology, bioinformatics, and validation experiments

**DOI:** 10.3389/fphar.2023.1079539

**Published:** 2023-03-01

**Authors:** Xiaoqing Xu, Yaping Yu, Li Yang, Bingshu Wang, Yonghao Fan, Banzhan Ruan, Xiaodian Zhang, Haofu Dai, Wenli Mei, Wei Jie, Shaojiang Zheng

**Affiliations:** ^1^ Department of Oncology of the First Affiliated Hospital & Cancer Institute, Hainan Medical University, Haikou, China; ^2^ Key Laboratory of Natural Products Research and Development from Li Folk Medicine of Hainan Province, Institute of Tropical Bioscience and Biotechnology, Chinese Academy of Tropical Agricultural Sciences, Haikou, China; ^3^ Key Laboratory of Emergency and Trauma of Ministry of Education & Key Laboratory of Tropical Cardiovascular Diseases Research of Hainan Province & Hainan Women and Children’s Medical Center, Hainan Medical University, Haikou, China

**Keywords:** pancreatic ductal adenocarcinoma, Dendrobium nobile, Dendrobin A, network pharmacology, bioinformatics, PLAU

## Abstract

**Background:** *Dendrobium nobile* (*D. nobile*), a traditional Chinese medicine, has received attention as an anti-tumor drug, but its mechanism is still unclear. In this study, we applied network pharmacology, bioinformatics, and *in vitro* experiments to explore the effect and mechanism of Dendrobin A, the active ingredient of *D. nobile*, against pancreatic ductal adenocarcinoma (PDAC).

**Methods:** The databases of SwissTargetPrediction and PharmMapper were used to obtain the potential targets of Dendrobin A, and the differentially expressed genes (DEGs) between PDAC and normal pancreatic tissues were obtained from The Cancer Genome Atlas and Genotype-Tissue Expression databases. The protein-protein interaction (PPI) network for Dendrobin A anti-PDAC targets was constructed based on the STRING database. Molecular docking was used to assess Dendrobin A anti-PDAC targets. PLAU, one of the key targets of Dendrobin A anti-PDAC, was immunohistochemically stained in clinical tissue arrays. Finally, *in vitro* experiments were used to validate the effects of Dendrobin A on PLAU expression and the proliferation, apoptosis, cell cycle, migration, and invasion of PDAC cells.

**Results:** A total of 90 genes for Dendrobin A anti-PDAC were screened, and a PPI network for Dendrobin A anti-PDAC targets was constructed. Notably, a scale-free module with 19 genes in the PPI indicated that the PPI is highly credible. Among these 19 genes, PLAU was positively correlated with the cachexia status while negatively correlated with the overall survival of PDAC patients. Through molecular docking, Dendrobin A was found to bind to PLAU, and the Dendrobin A treatment led to an attenuated PLAU expression in PDAC cells. Based on clinical tissue arrays, PLAU protein was highly expressed in PDAC cells compared to normal controls, and PLAU protein levels were associated with the differentiation and lymph node metastatic status of PDAC. *In vitro* experiments further showed that Dendrobin A treatment significantly inhibited the proliferation, migration, and invasion, inducing apoptosis and arresting the cell cycle of PDAC cells at the G2/M phase.

**Conclusion:** Dendrobin A, a representative active ingredient of *D. nobile*, can effectively fight against PDAC by targeting PLAU. Our results provide the foundation for future PDAC treatment based on *D. nobile*.

## Introduction

Pancreatic ductal adenocarcinoma (PDAC) is a highly aggressive malignancy with poor clinical prognosis and treatment outcomes, and its incidence and mortality rates have been increasing (GBD, 2017 Pancreatic Cancer Collaborators, 2019). Near 80%–90% of patients who suffered from PDAC had unresectable tumors or metastatic disease at the time of the first diagnosis, with a 5-year overall survival (OS) rate of 10%, despite the development of the radical surgical treatment. Although gemcitabine is a recognized first-line chemotherapy drug for PDAC, it is still unclear whether there is an advantage for survival (Zhang et al., 2022). Therefore, new cancer treatments are urgently needed for the treatment of metastatic or incurable PDAC (Von Hoff et al., 2013; Chen et al., 2020).

Cancer cachexia is a multifactorial combination of disease symptoms characterized by muscle wasting, which can lead to significant weight loss and affect patients’ quality of life, treatment-tolerant response, and survival (Vaughan et al., 2013). Freire et al. (2020) reported that variants in cachexia-inducible factors and their associated regulatory genes had the highest frequency in pancreatic cancer patients, and the expression levels of key cachexia genes had predictive prognostic value in pancreatic cancer. Meanwhile, specifically targeting PLA2G7 can alleviate cachexia levels in tumor-bearing mice (Morigny et al., 2021). These findings suggest that identification of cachexia-related factors and targeted intervention of cachexia play an important role in the treatment of malignant tumors, such as pancreatic cancer.

The use of traditional Chinese medicine (TCM) in pancreatic cancer treatment is gradually attracting the attention of clinicians. *Dendrobium nobile* (*D. nobile*), as a typical representative of TCM, has recently been reported in the field of anti-tumor drugs. Reports have increasingly shown that bibenzyl compounds extracted from *D. nobile* displayed promising effects against cancers derived from the lung (Losuwannarak et al., 2020; Putri et al., 2021), skin (Cardile et al., 2020), bladder (Zhu et al., 2019), liver (Chen et al., 2017), and breast (Yu et al., 2018). Dendrobin A (4,5-dihydroxy-3,3′-dimetheoxybibenzyl) is a new bibenzyl, and there is a lack of studies related to its role in pancreatic cancer; therefore, further elucidation of the anti-pancreatic cancer effect of Dendrobin A and other Dendrobium species is of great significance for the development of TCM anti-tumor therapy.

Network pharmacology is a novel approach that integrates systems biology and bioinformatics methods to predict drug targets using biological networks and big data technologies (Hopkins, 2008; Nogales et al., 2022), which has transformed the drug mechanism of action from a single-target, single-drug model to a network-target, multi-component therapeutic model, and has shown exciting results, especially in the field of TCM target prediction (Zhang et al., 2019). In this study, we integrated PDAC transcriptome expression data by network pharmacology technology and preferentially selected drug targets based on biological network characterization and survival analysis. Additionally, we performed *in vitro* experiments to validate the effects of Dendrobin A extracted from *D. nobile* on PDAC cells. Our results provide novel insights into Dendrobin A in treating PDAC and support the use of *D. nobile* in conventional medicine.

## Materials and methods

### Extraction and isolation of *D. nobile* gradient Dendrobin A

Dendrobin A was extracted and isolated from the *D. nobile* plant as per previously described protocols (Cao et al., 2021). Briefly, the air-dried stems of *D. nobile* (13.0 kg) were powdered and extracted with 95% EtOH three times. Then, the concentrated ethanolic extract (716.2 g) was suspended in H_2_O and successively partitioned with petroleum ether, EtOAc, and *n*-BuOH. Subsequently, the EtOAc extract (87.9 g) was fractionated by silica gel vacuum liquid column chromatography (CC) eluted with petroleum ether-EtOAc (20:1, 10:1, 5:1, 2:1, 1:1, 0:1, v/v) to generate 16 fractions (Fr.1–16). Fr.8 was separated by Rp-18 CC eluted with methanol-H_2_O (3:7→1:0) to obtain ten subfractions (Fr.8-1–Fr.8-10). Fr.8-6 was applied to the Sephadex LH-20 with chloroform-methanol (1:1) as eluent to produce three subfractions (Fr.8-6-1–Fr.8-6-3). Fr.8-6-1 was purified by silica gel CC eluted with petroleum ether-acetone (20:1→0:1) to give compound Dendrobin A.

### Screening potential targets of Dendrobin A

To identify the potential targets of Dendrobin A, we first collected the drug compound information from PubChem (https://pubchem.ncbi.nlm.nih.gov/) with PubChem CID 44418770. The Dendrobin A canonical SMILES ‘COC1 = CC = CC (=C1) CCC2 = CC(=C(C(=C2)OC)O)O’ was submitted into the drug target predictions web server SwissTargetPrediction (Gfeller et al., 2014) and PharmMapper (Wang et al., 2017). After redundancy analysis and standardization, 353 well-reported pharmacological targets of Dendrobin A were obtained.

### Differential expression genes (DEGs) of PDAC patients

Due to the limitation of adjacent normal samples in The Cancer Genome Atlas (TCGA) datasets, we performed DEG analysis by integrating the transcriptome of PDAC tumor samples in TCGA and normal pancreas tissues in the Genotype-Tissue Expression (GTEx) datasets according to the algorithm proposed in a previous report (Vivian et al., 2017). Data were obtained from the “TCGA TARGET GTEx” cohort in UCSC Xena (http://xena.ucsc.edu/), including sequencing counts and Transcripts Per Million (TPM) normalized expression matrices. All data were normalized and batch-corrected; the datasets used in this study included 178 cancer and 167 normal samples. The R package edgeR 3.30.3 (Robinson et al., 2010) was applied for DEGs analysis based on the gene read counts matrix. Only genes with |log2FC| > 2 and FDR <0.05 were considered significantly differentially expressed. An external dataset of PDAC was also downloaded from the GEO database (https://www.ncbi.nlm.nih.gov/geo/) with accession number GSE62452 (Yang et al., 2016), which included 69 pancreatic tumor and adjacent non-tumor tissues to validate the DEGs and clinical significance.

### Construction and topological analysis of protein-protein interaction (PPI) network

The putative target genes of Dendrobin A and DEGs in PDAC samples were overlapped to identify the shared target genes for Dendrobin A in treating PDAC. These essential genes were next input into the STRING V11.5 (Szklarczyk et al., 2019) to construct the PPI network with default parameters. We performed a Pearson correlation analysis for each gene pair of the PPI network in PDAC samples to obtain the actual relationships in the tumor context. Correlation coefficients above 0.2 and *p*-value <0.05 were identified as significant. The PDAC-related PPI network was visualized using Cytoscape V3.7.2 (Shannon et al., 2003). The MCODE plugin was applied to identify the network modules. The PPI network’s degree distribution, clustering coefficient, and characteristic path length were analyzed using R package igraph 1.2.6. To explore the potential biological function of the PPI network, we input the genes to Metascape (https://metascape.org/gp/index.html#/main/step1) with the setting of species (“*Homo sapiens*”) (Zhou et al., 2019).

### Survival prognosis analysis

The survival analysis of OS for essential genes of PDAC tumors in TCGA cohorts was performed through GEPIA2 (http://gepia2.cancer-pku.cn/) (Tang et al., 2019). The median expression of genes was used to divide the patients into high-expression and low-expression groups. Then, the OS of these groups was compared by log-rank test.

### Estimation of the association between genes and cachexia

The information on 25 cachexia-inducing factors (CIFs) was collected from a previous study (Freire et al., 2020). The single-sample GSEA (ssGSEA) algorithm (Barbie et al., 2009) was applied to estimate the cachexia score for each sample. Wilcoxon’s rank sum tests evaluated the cachexia score between PDAC in TCGA and normal samples in GTEx. Pearson correlation analysis was performed to estimate the association between the expression level of genes and cachexia score.

### Molecular docking

In this study, the drug-protein binding patterns were predicted by molecular docking, which was performed using AutoDock Vina 1.1.21 software (The Scripps Research Institute, La Jolla, CA, United States) (Trott and Olson, 2010). Before the start of docking, the PLAU protein crystal structure was obtained from the PDB database with the protein search number 6AG7. The 3D structure of Dendrobin A was obtained from the PubChem database download, and energy minimization of the small molecule was performed using the MMFF94 force field. Before the start of formal docking, the protein was prepared using PyMol 2.5.2 software, including dehydrogenation, dehydration molecules, and non-liganded small molecules. The docking box was then defined to wrap the protein activity pocket. Then, the small molecules in PDB format and the receptor proteins were converted to PDBQT format using ADFRsuite 1.02 (Ravindranath et al., 2015). Finally, the docking work was performed, visualized, and analyzed using PyMol 2.5.2.

### Assessment of Dendrobin A on cell proliferation by cell counting Kit-8 (CCK-8) assay

The human PDAC cell lines Panc-1 and Aspc-1, as well as the normal pancreatic cell line HPDE6-C7, were purchased from the Shanghai Chinese Academy of Sciences Cell bank. All cells were tested by short tandem repeat to confirm that there was no cross-contamination, and routine *mycoplasma* and *chlamydia* contamination tests were performed before the experiments. Cells were cultured in Dulbecco’s modified eagle medium (HyClone, Beijing, China) supplemented with 10% fetal bovine serum (HyClone), 100 μg/mL streptomycin, and 100 U/mL penicillin (Beyotime, Nanjing, China), at 37°C in 5% CO_2_. Dendribin A was dissolved in dimethyl sulfoxide (DMSO), and the concentration of DMSO was <0.1%. The proliferation and cytotoxicity of three pancreatic cell lines were measured by a cell counting kit (CCK-8, Beyotime); the cells were seeded into a 96-well plate (2,000 cells per well) and incubated overnight. Cells were treated with 10, 20, 30, 40, 50, 60, 70, 80, 90, and 100 µM of Dendrobin A for 24 h at 37°C. After the CCK-8 reagent was added and incubated for 1 h, the optical density was detected at the 450 nm wavelength. Five replicate wells were used for each group, and experiments were repeated three times.

### Clone formation assay

PDAC cells at a density of 3,000 cells/well were seeded into 6-cm dishes overnight. The cells were treated with a medium containing Dendrobin A or DMSO for 24 h, and then the medium was replaced with Dendrobin A-free completed medium and cultured for 15 days. The culture medium was discarded, and then cells were washed twice with PBS, fixed in methanol for 20 min, and air dried after discarding methanol. The cells were stained with 0.3% crystal violet solution for 15 min and washed gently with ddH_2_O to remove the staining solution. The number of clones with >50 cells was counted after photographing. Statistical analysis was performed using Image J software (version 1.8.0). Experiments were repeated three times.

### Wound healing assay

PDAC cells were seeded on 12-well plates to reach the monolayer, and a linear wound was formed by scraping with a 200 μL micropipette tip, and the cells were treated with Dendrobin A for 24 h. The cell scratch area was observed with a ×20 microscope objective and photographed, and the wound distance was used to assess the cell migration rate. The degree of wound healing was calculated by analysis with Image J software (version 1.8.0). Experiments were repeated three times.

### Invasive and migration assay

The ability of PDAC cells to invade and migrate was assayed using the Transwell method. Matrigel gel (BD, China, Shanghai) and serum-free medium were diluted at a ratio of 1:8, and 100 μL per well was added in the upper chamber of the Transwell (Corning, NC, United States) and incubated at 37°C for 3 h. The migration assay was performed without Matrigel gel, and the procedure was the same. 4 × 10^4^ Aspc-1 cells and 1 × 10^5^ Panc-1 cells were loaded into the upper chamber with 200 µL of serum-free medium, while medium containing 20% serum was added to the lower chamber after 24 h of incubation. The upper chamber was fixed in 4% paraformaldehyde and stained with 0.1% crystal violet. Invaded or migrated cells were counted and photographed, and differences in the number of cells that penetrated the membrane represented the altered cell motility. Statistical analysis was performed using Image J software (version 1.8.0). Experiments were repeated three times.

### Flow cytometry analysis of cell cycle and apoptosis

PDAC cells were added in 6-well plates allowing recovery overnight, then Dendrobin A was added for 24 h, and the cells were harvested for cell cycle and apoptosis analysis. PDAC cells were stained with propidium iodide (PI) for cell cycle analysis. Cell Cycle Assay Kit-PI/RNase staining (BD, China) was followed by the detection of cell ratios in the G0/G1, S, and G2/M phases. For apoptosis, PDAC cells were stained with Annexin V fluorescein isothiocyanate (FITC) and PI using the Annexin V-FITC Apoptosis Detection Kit (BD, China). Three wells were used for each group. Experiments were repeated three times.

### Tissue microarrays and immunohistochemical staining

Human tissue microarrays of PDAC (#HPanA180Su03, Shanghai Outdo Biotech Company, China) were used to detect the expression of PLAU protein. Tissue arrays include 81 cancer tissues and 81 paracancerous tissues from surviving PDAC patients. Immunohistochemical staining (IHC) was performed using an antibody against PLAU (1:500 dilution, #17968-1-AP, Proteintech, Wuhan, China). The immunohistochemical staining protocol was performed according to the literature (Bai et al., 2022). The scores of PLAU were calculated briefly as staining intensity: 0, no staining; 1, light yellow; 2, brown; and 3, dark brown; staining positive rate: 0%–100%. The total score was calculated as follows: “staining intensity score” × “staining positive rate” ×10. The mean value of a total score of 15.75 for all PDAC samples was used as the grouping criterion, and samples with a score of ≥15.75 belonged to the high-expression group, while samples with a score of <15.75 were included in the low-expression group.

### Western blot

Total proteins were extracted using RIPA buffer (Thermo Fisher Scientific, MA, United States). A total of 30 μg proteins was subjected to 10% Sodium dodecyl sulfate-polyacrylamide gel electrophoresis (SDS-PAGE), and proteins were transferred to a polyvinylidene fluoride membrane (Merk Millipore, Darmstadt, Germany). The membranes were blocked in 5% bovine serum albumin and incubated with primary antibodies anti-PLAU (1:2,000, Proteintech) and β-actin (1:2000, #66009-1-Ig, Proteintech) at 4°C overnight. The membranes were incubated with HRP-conjugated IgGs (1:1,000, #7074, CST, China, Shanghai) for 1 h at room temperature, and the protein was detected using enhanced chemiluminescence (Bio-Rad, China, Shanghai). Protein bands were analyzed using Image J software (version 1.8.0). Experiments were repeated three times.

### Statistical analysis

For network pharmacology and bioinformatics, the statistical analyses were performed using R software. The associations between clinical features and PLAU expression were evaluated using the Wilcoxon signed-rank test and the chi-square test. Clinical features related to OS in PDAC patients were identified using the Kaplan-Meier method. For *in vitro* experiments, data were shown as mean ± SD. Statistical analysis was performed with GraphPad Prism 8.0 software, and the significance was analyzed with Student’s t-test or ANOVA. *p* < 0.05 was considered statistically significant.

## Results

### Extraction, isolation, and identification of Dendrobin A

**FIGURE 1:**
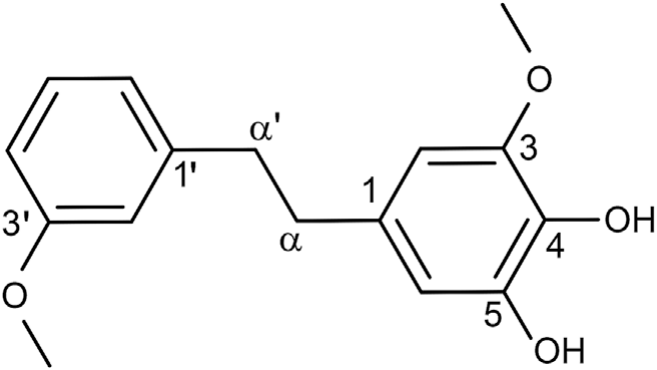
The structure of Dendrobin A.

According to our previously described protocols (Cao et al., 2021), we successfully obtained 548.4 mg Dendrobin A from 13.0 kg *D. nobile*. The structure of Dendrobin A is shown in Figure 1. In addition, the ^1^H NMR (500 MHz) and ^13^C NMR (125 MHz) data of Dendrobin A in CDCl3 were included in Table 1.

**TABLE 1:** 1H NMR (500 MHz) and 13C NMR (125 MHz) data of Dendribin A in CDCl_3_.

No.	*δ*_H_ (*J* in Hz)	*δ*_C_, type
1	—	132.9, C
2	6.25, s	103.6, CH
3	—	146.8, C
4	—	130.6, C
5	—	143.9, C
6	6.47, br s	108.7, CH
α	2.82, m	38.3, CH_2_
α′	2.82, m	37.9, CH_2_
1′	—	143.5, C
2′	6.73, s	114.4, CH
3′	—	159.7, C
4′	6.75, d (*J* = 7.6 Hz)	111.4, CH
5′	7.20, t (*J* = 7.7 Hz)	129.4, CH
6′	6.79, br d (*J* = 7.5 Hz)	121.1, CH
3-OCH_3_	3.83, s	56.2, CH_3_
3ʹ-OCH_3_	3.79, s	55.3, CH_3_

### Identification and analysis of Dendrobin A potential targets in PDAC

**FIGURE 2:**
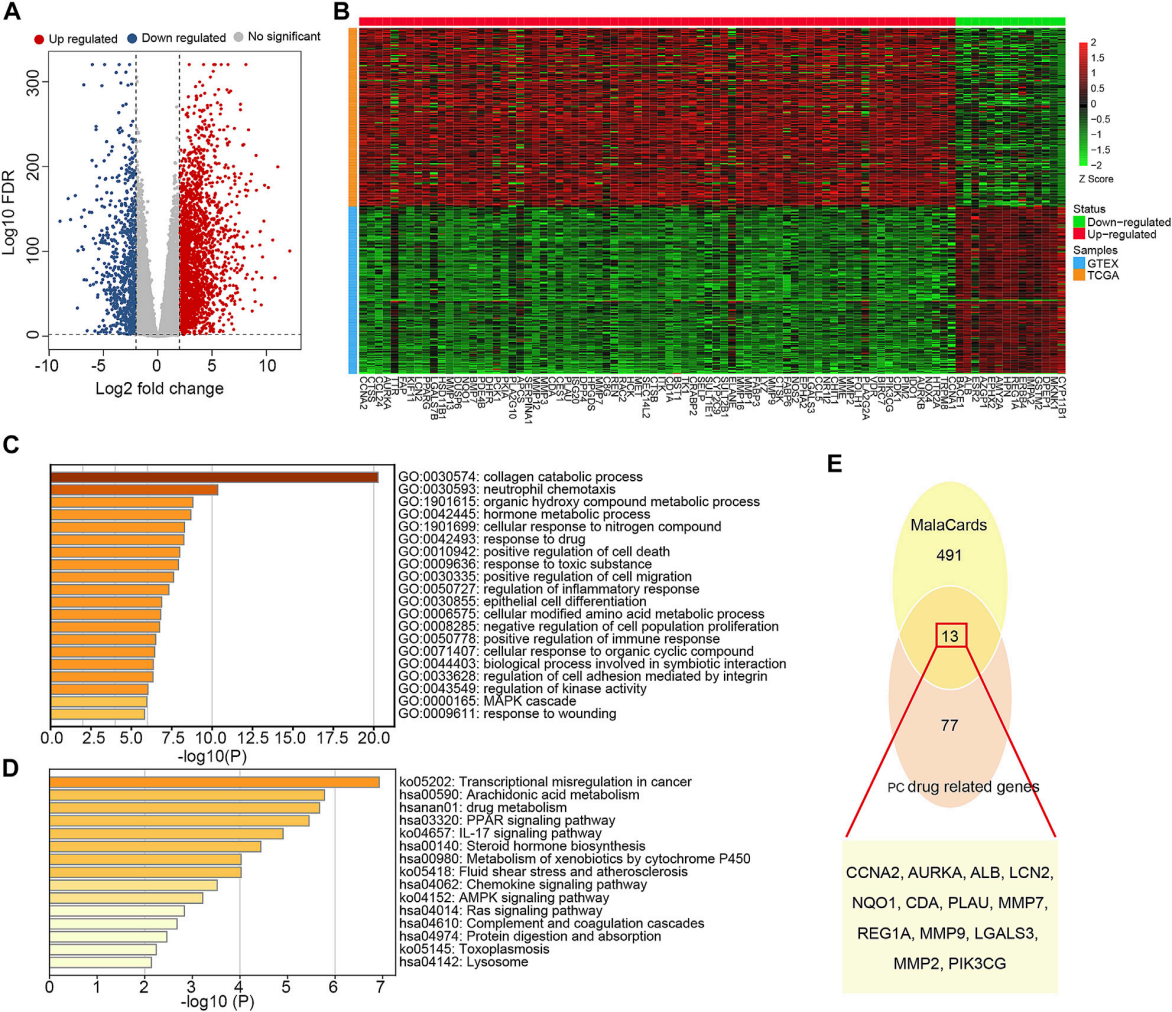
Identification and functional annotation of the overlapped targets of pancreatic ductal adenocarcinoma (PDAC) and Dendrobin A. **(A)** Volcano plot of differentially expressed genes related to PDAC; red dots represent upregulated genes, and blue dots represent downregulated genes. **(B)** Heat map of Dendrobin A target genes involved in PDAC; red boxes represent upregulated genes, and green boxes represent downregulated genes. **(C)** The biological process of Dendrobin A target genes involved in PDAC in GO annotation. **(D)** KEGG pathway enrichment of Dendrobin A target genes involved in PDAC. **(E)** Dendrobin A target genes involved in PDAC based on MalaCards.

Based on SwissTargetPrediction and PharmMapper databases, 353 pharmacological targets of Dendrobin A were identified. To explore the expression pattern of these putative drug targets in a tumor context, we performed DEG analysis by integrating the transcriptome of pancreas tumor in TCGA and normal pancreatic samples in GTEx datasets. We identified 3,131 DEGs, comprising 2,358 upregulated and 773 downregulated genes in PDAC samples (Figure 2A). By overlapping 3,131 DEGs and 353 drug targets, we found 90 genes related to Dendrobin A treatment and expressed differently in PDAC samples. Among them, 76 were upregulated, and 14 were downregulated (Figure 2B). Further functional enrichment analysis indicated that these genes were significantly enriched in several metabolism-related biological processes like collagen catabolic, organic hydroxyl compound metabolic process, and response to the drug (Figure 2C). For KEGG pathways, cancer-related pathways like transcriptional misregulation, PPAR signaling, and drug metabolism were significantly enriched (Figure 2D). Next, we obtained 504 genes related to PDAC from MalaCards and intersected with the 90 essential genes mentioned above. We identified 13 shared genes, namely, *CCNA2*, *AURKA*, *ALB*, *LCN2*, *NQO1*, *CDA*, *PLAU*, *MMP7*, *REG1A*, *MMP9*, *LGALS3*, *MMP2*, and *PIK3CG* (Figure 2E).

### Dendrobin A-related PPI network

**FIGURE 3:**
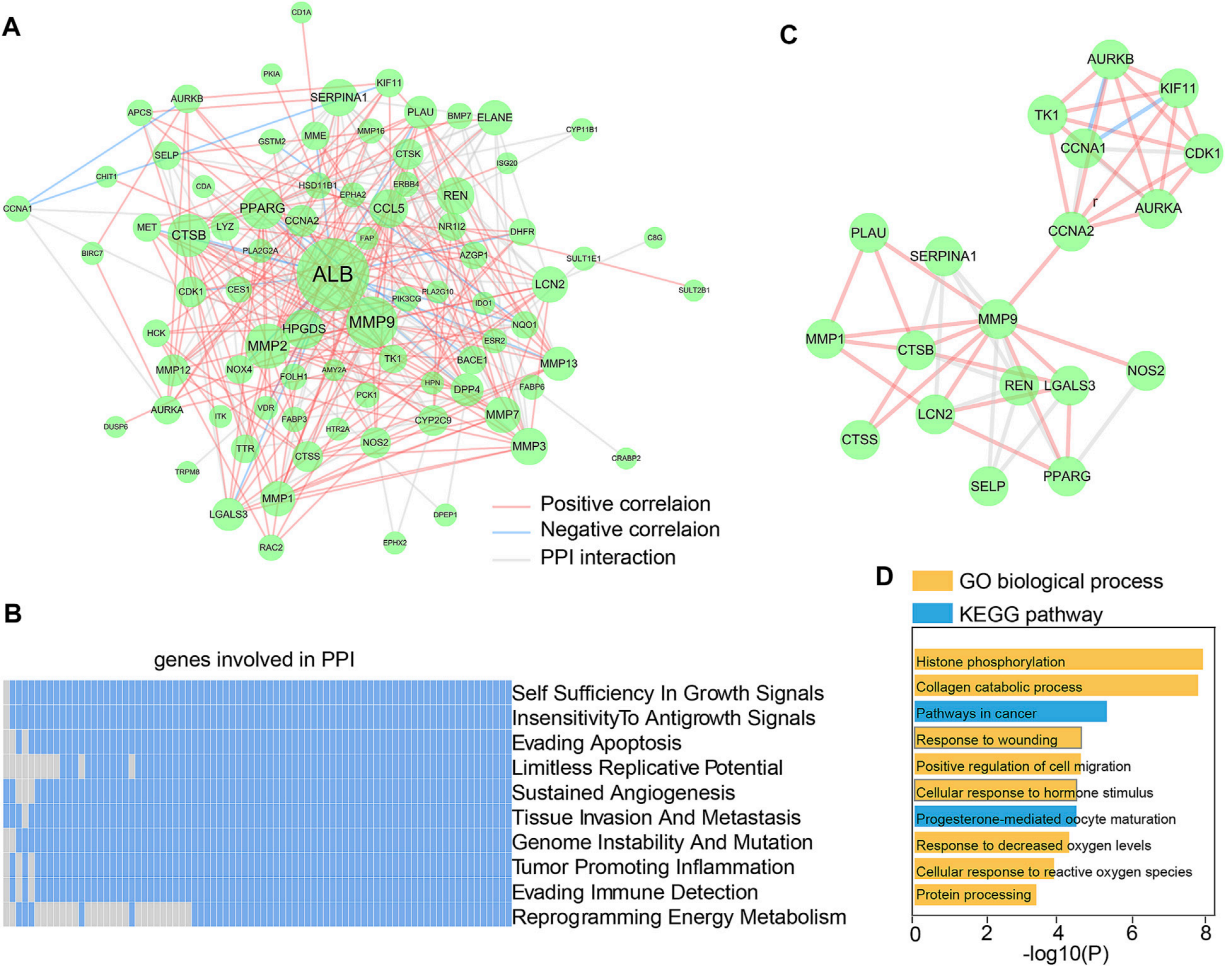
Construction and functional analysis of Dendrobin A-associated PPI network. **(A)** Dendrobin A-related PPI network in PDAC; red lines represent positively interacting genes in PDAC, blue lines represent negatively interacting genes in PDAC, and the node sizes represent connectivity. **(B)** Association of genes in PPI network with ten cancer hallmarks; blue indicates the positive relation to cancer hallmarks, and gray indicates non-relation. **(C)** Key network module based on MCODE. **(D)** Module function enrichment.

The 90 essential Dendrobin A-related DEGs were input into STRING to obtain the interactions of proteins. As a result, a PPI network containing 81 nodes and 299 edges was constructed. Through co-expression analysis and based on the threshold of |Pearson r| > 0.2 & *p* < 0.05, we found 57.86% (173/299) positive correlation edges and 7.02% (21/299) negative correlation edges in PDAC samples (Figure 3A). We utilized the hierarchical model that linked genes, biological processes, and previously described cancer hallmarks to determine the association between the PPI network and tumorigenesis (Poulia et al., 2020). Many genes in the PPI network were associated with multiple cancer hallmarks (Figure 3B). These results suggest that Dendrobin A-related genes play essential roles in cancer processes. Through topological feature analysis, we found that the PPI network displayed scale-free distribution with *R* ^2^ = 0.682 (Supplementary Figure S1A), suggesting that the network showed scale-free characteristics. Moreover, the PPI network’s clustering coefficient and characteristic path length were significantly increased when compared with random networks, as expected for reduced global efficiency and module characteristics (*p*-value <0.001, Supplementary Figures S1B, C). Therefore, a critical PPI network module with 19 genes was extracted through the MCODE plugin in Cytoscape software (score: 5.556, Figure 3C). The functional enrichment results showed that this module was strongly associated with histone phosphorylation, collagen catabolic process, and pathways in cancer (Figure 3D).

### Expression and survival significance of Dendrobin A-related genes in PDAC

**FIGURE 4:**
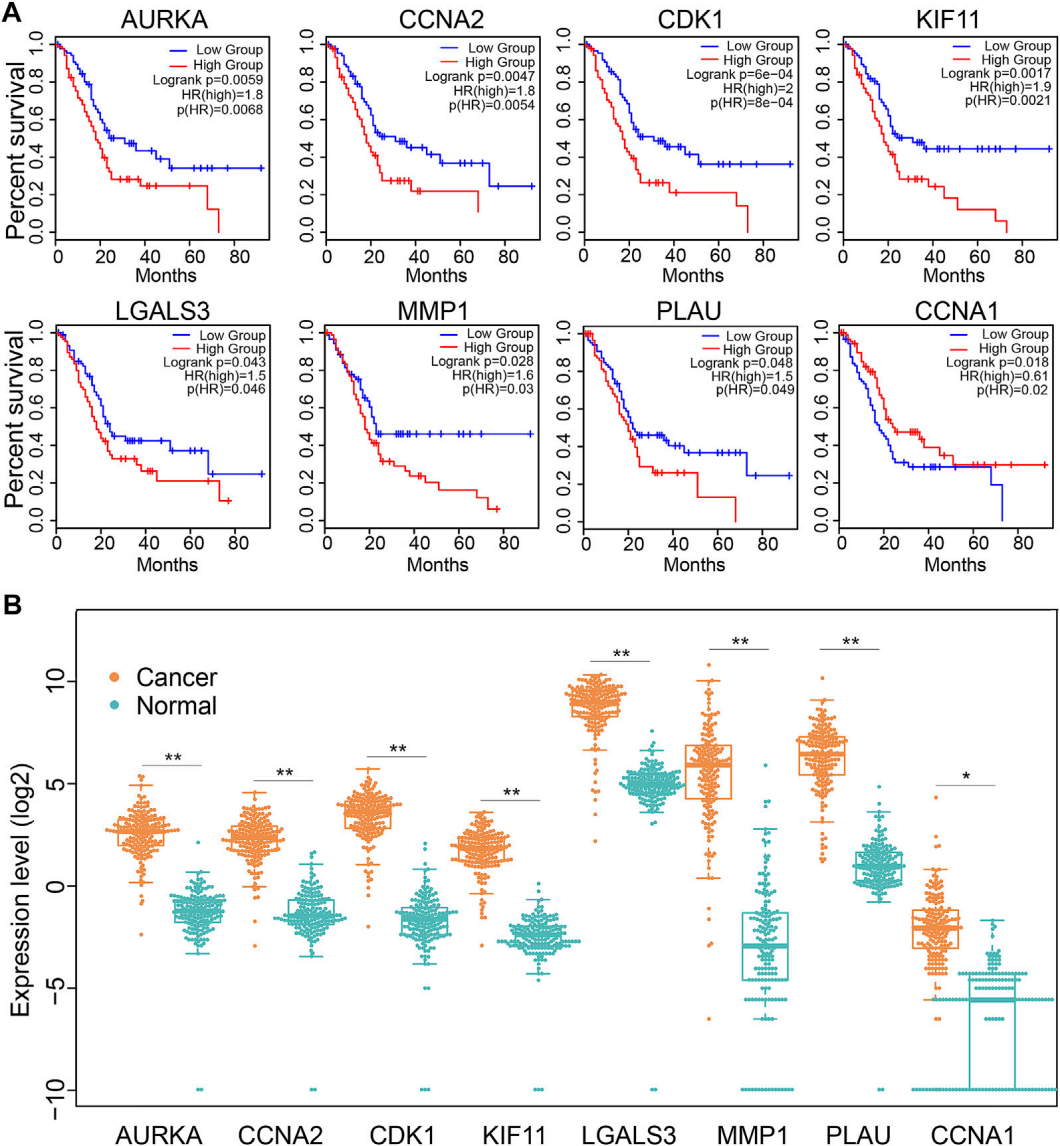
The expression and association of key Dendrobin A-related genes with OS in the PDAC cohort. **(A)** Overall survival analysis of key Dendrobin A-related genes in PDAC. **(B)** Expression of key Dendrobin A-related genes in PDAC and normal pancreatic tissues. Wilcoxon’s rank sum tests, *p < 0.05, **p < 0.01.

We next explored the significance of 19 Dendrobin A-related genes in the survival of PDAC patients. We found that patients with higher expression of *AURKA*, *CCNA2*, *CDK1*, *KIF11*, *LGALS3*, *MMP1,* and *PLAU* had worse OS in PDAC patients (HR > 1 and log-rank *p* < 0.05). At the same time, *CCNA1* indicated a favorable prognosis in PDAC (HR < 1 and log-rank *p* < 0.05, Figure 4A), and the remaining 11 genes showed no significance with OS of PDAC (data not shown). Furthermore, the expression levels of these eight OS-related genes were displayed (Figure 4B). Therefore, these eight OS-related genes, except *CCNA1,* may play oncogenic roles in PDAC.

### Association of Dendrobin A-related genes with cachexia in PDAC

**FIGURE 5:**
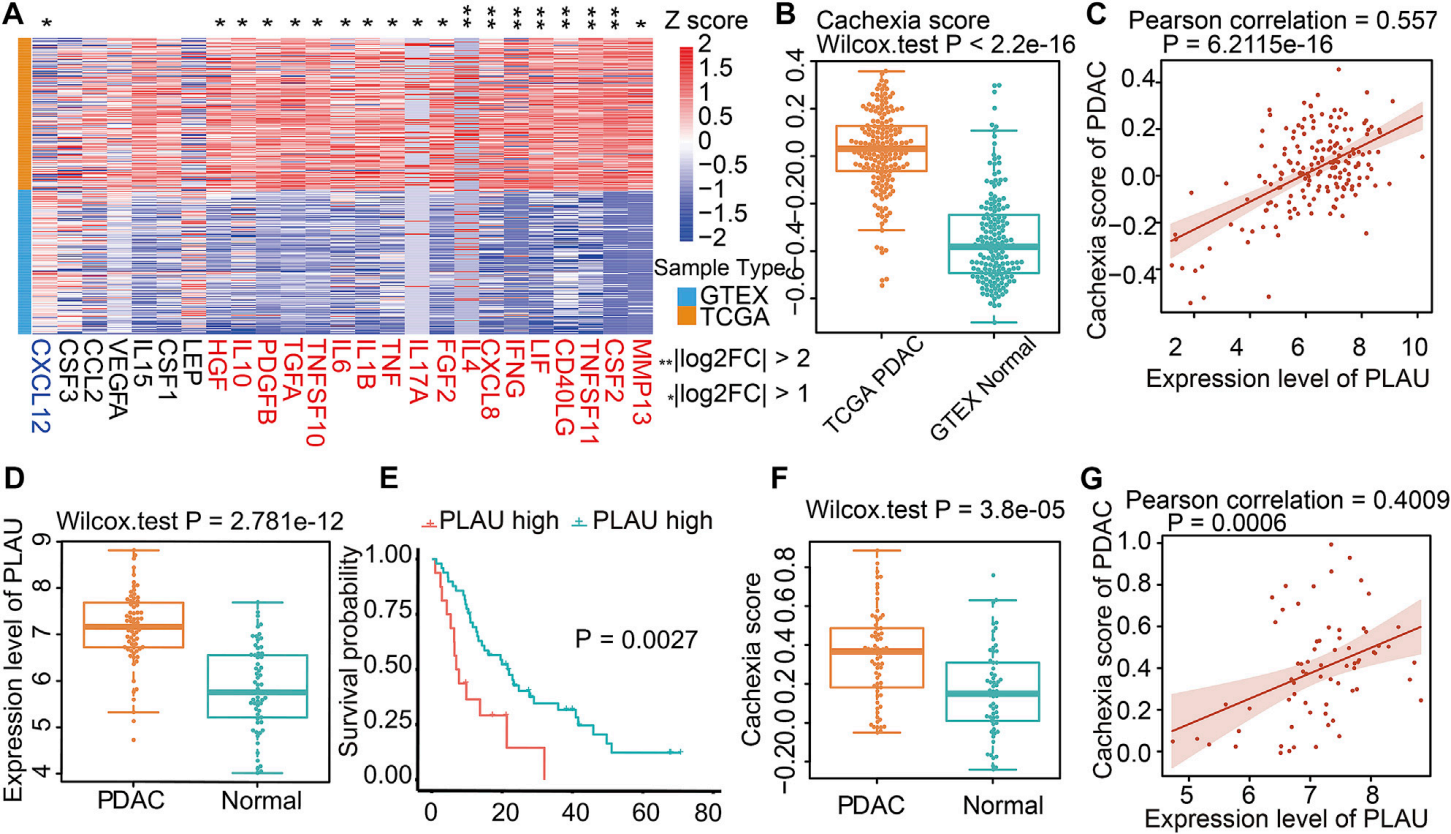
Cachexia-inducing factors in PDAC and validation. **(A)** Enrichment of Cachexia-inducing factors in PDAC based on TCGA and GTEX databases. Genes in red indicate a positive association with cachexia. **(B)** Comparison of cachexia scores for genes in red shown in Figure 5A for PDAC and normal tissue. **(C)** Correlation of PLAU gene with cachexia score in PDAC. **(D–G)** Validation of the expression, correlation PLAU in PDAC, and cachexia using external data from GEO dataset GSE62452.

Since cachexia is a classic feature of PDAC patients (Freire et al., 2020), we next explored whether the dysregulation of Dendrobin A-related genes was associated with cachexia in PDAC patients. DEG analysis showed that 18 of 25 cachexia-induced factors were upregulated in PDAC samples (Figure 5A). In addition, the cachexia score of cancerous pancreatic tissues was significantly higher than that of normal pancreatic tissues (Figure 5B). We thus evaluated the associations between risky prognostic genes and cachexia in PDAC patients. As a result, the expression level of PLAU was significantly positively correlated with the cachexia score (Figure 5C). Consistent with this finding, a similar upregulated expression pattern, prognostic risk, and the positive correlation with cachexia of *PLAU* levels in another PDAC dataset were observed (GSE62452, Figures 5D–G). We analyzed the association between *PLAU* mRNA levels and PDAC patients’ clinical parameters, including age, gender, stage, and nodal metastasis status. The results showed that *PLAU* was differentially expressed between stage 1 and stage 2 (Supplementary Figure S2).

### Molecular docking

**FIGURE 6:**
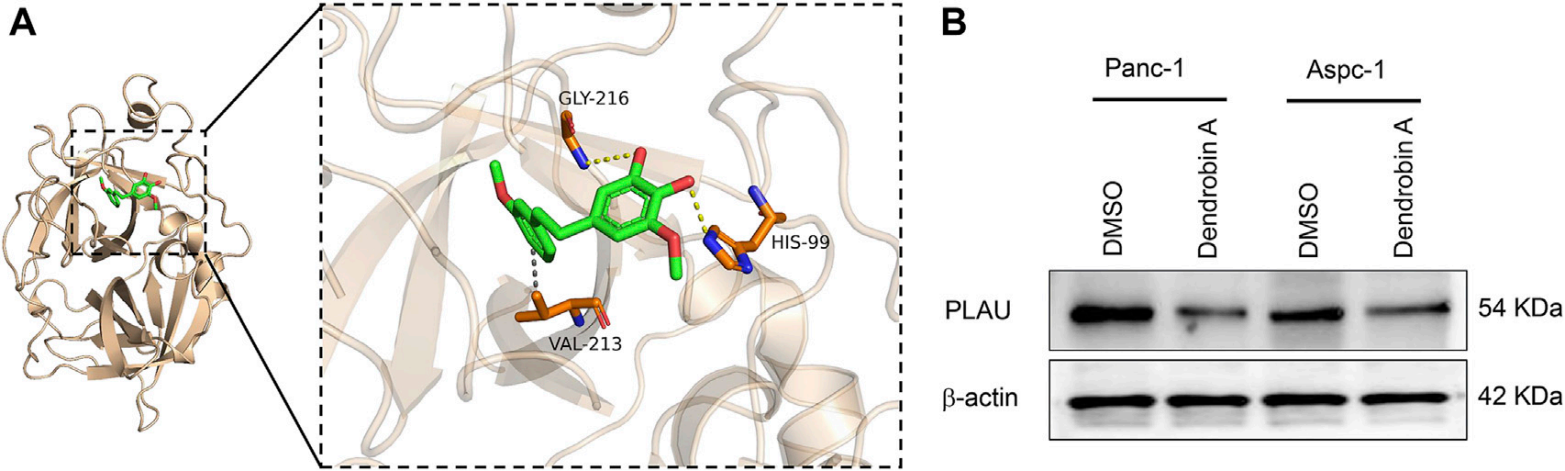
Molecular docking of Dendrobin A and PLAU. **(A)** The representative images for molecular docking of Dendrobin A and PLAU. Dendrobin A (green) binds with PLAU (orange) using hydrophobic interaction and hydrogen (dashed line). **(B)** Dendrobin A inhibiting PLAU protein expression in PDAC cells assessed by western blotting.

Molecular docking is a convenient and effective means to explore the interaction of small molecules with their targets (Trott and Olson, 2010; Ravindranath et al., 2015); we used Vina 1.1.21 software to perform a molecular docking study of Dendrobin A with PLAU proteins. Interestingly, Dendrobin A is bound within the PLAU active pocket (score: −6.9 kcal/mol) (Figure 6A). In addition, western blot results showed that Dendrobin A treatment reduced the expression level of PLAU protein in PDAC cells (Figure 6B).

### PLAU expression and association with clinical parameters in PDAC patients

**FIGURE 7:**
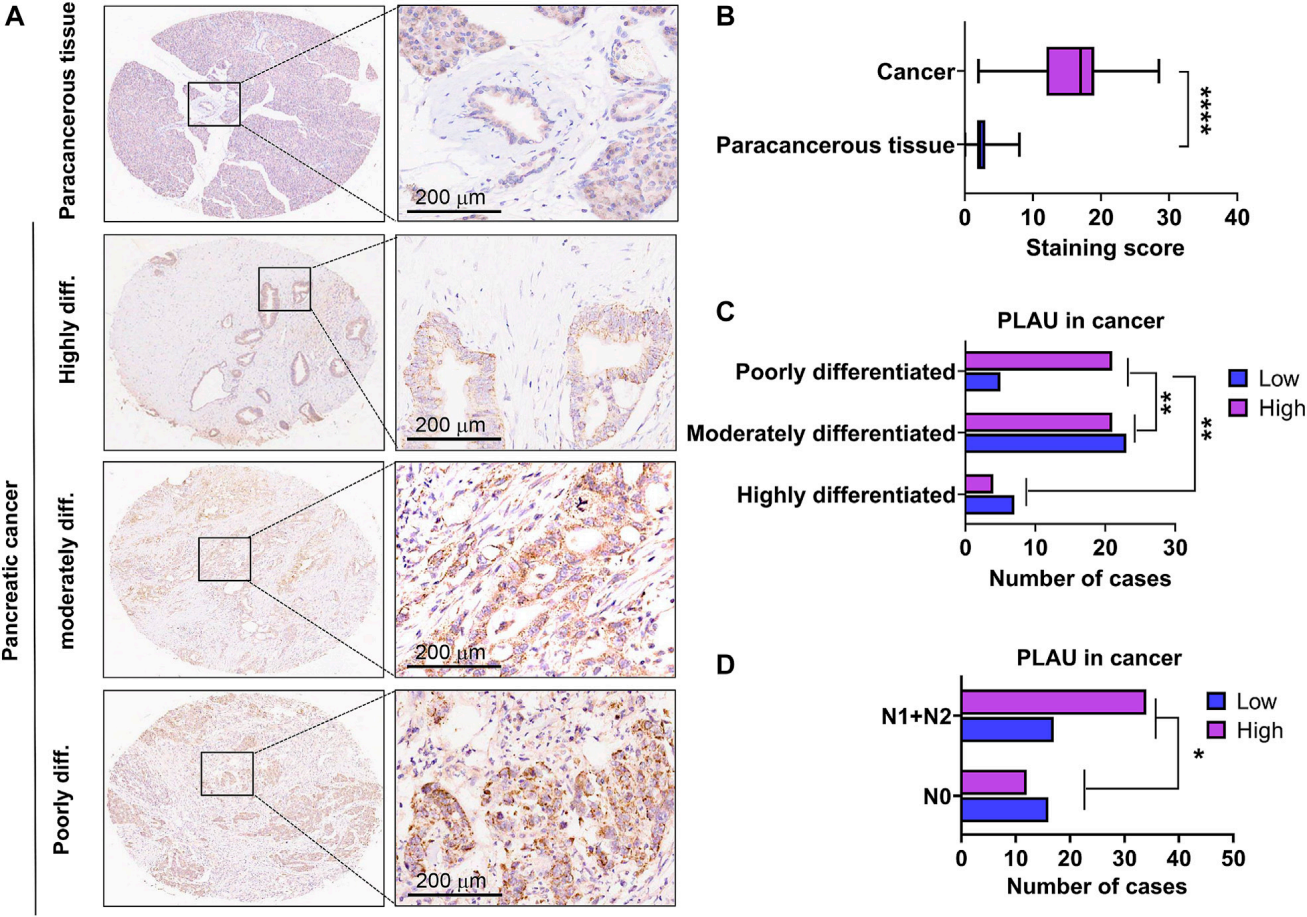
Expression levels of PLAU protein in PDAC and paraneoplastic tissues. **(A)** Representative images of PLAU protein in PDAC and control tissues by IHC staining. Bars = 200 μm. **(B)** Quantitative results of PLAU protein in PDACs and controls, ****p < 0.0001. **(C)** Correlation between PLAU protein expression in PDAC and tumor differentiation status of patients, **p < 0.01. **(D)** Correlation between PLAU protein expression in PDAC and lymph node metastatic status of patients, *p < 0.05.

We further examined PLAU protein expression in clinical PDAC samples by IHC. Commercial tissue arrays with 81 cases of PDAC and paired paraneoplastic tissues were used. The results showed that PDAC PLAU protein levels were significantly enhanced compared to paraneoplastic tissues (Figure 7A). Further analysis showed that higher PLAU protein expression in PDAC was positively correlated with the differentiation and lymph node metastasis status of PDAC (Figures 7B, C). Interestingly, PLAU protein expression in PDAC was associated with gender but not age, T classification, M classification, TNM stage, tumor size, and vascular and nerve invasion (Table 2).

**TABLE 2:** Relationship between PLAU protein expression and clinical pathological parameters of PDAC.

Clinical parameters	n	PLAU level		*p-*value
		Low	High	
Age	—	—	—	—
<60	43	17	26	0.478
≥60	38	18	20	
Gender	—	—	—	—
Female	47	15	32	0.016^a^
Male	34	20	14	
Differentiation	—	—	—	—
High	11	7	4	0.009^a^
Moderate	44	23	21	
Low	26	5	21	
T classification^b^	—	—	—	—
T1 + T2	38	20	18	0.081
T3 + T4	42	14	28	
N classification^b^	—	—	—	—
N0	28	16	12	0.040^a^
N1 + N2	51	17	34	
M classification	—	—	—	0.206
M0	59	28	31	
M1	22	7	15	
TNM stage	—	—	—	—
Ι + II	46	28	18	0.108
III + IV	35	15	20	
Tumor size^b^	—	—	—	—
≤5 cm	60	25	35	0.515
>5 cm	20	10	10	
Vascular invasion^b^	—	—	—	—
No	45	21	24	0.454
Yes	34	13	21	
Nerve invasion^b^	—	—	—	—
No	27	11	16	0.763
Yes	52	23	29	

^a^ Significance as indicated.

^b^ Data for 1 or 2 cases were missing.

### *In vitro* validation of Dendrobin A on the proliferation, cycle, apoptosis, and migration in PDAC cells

**FIGURE 8:**
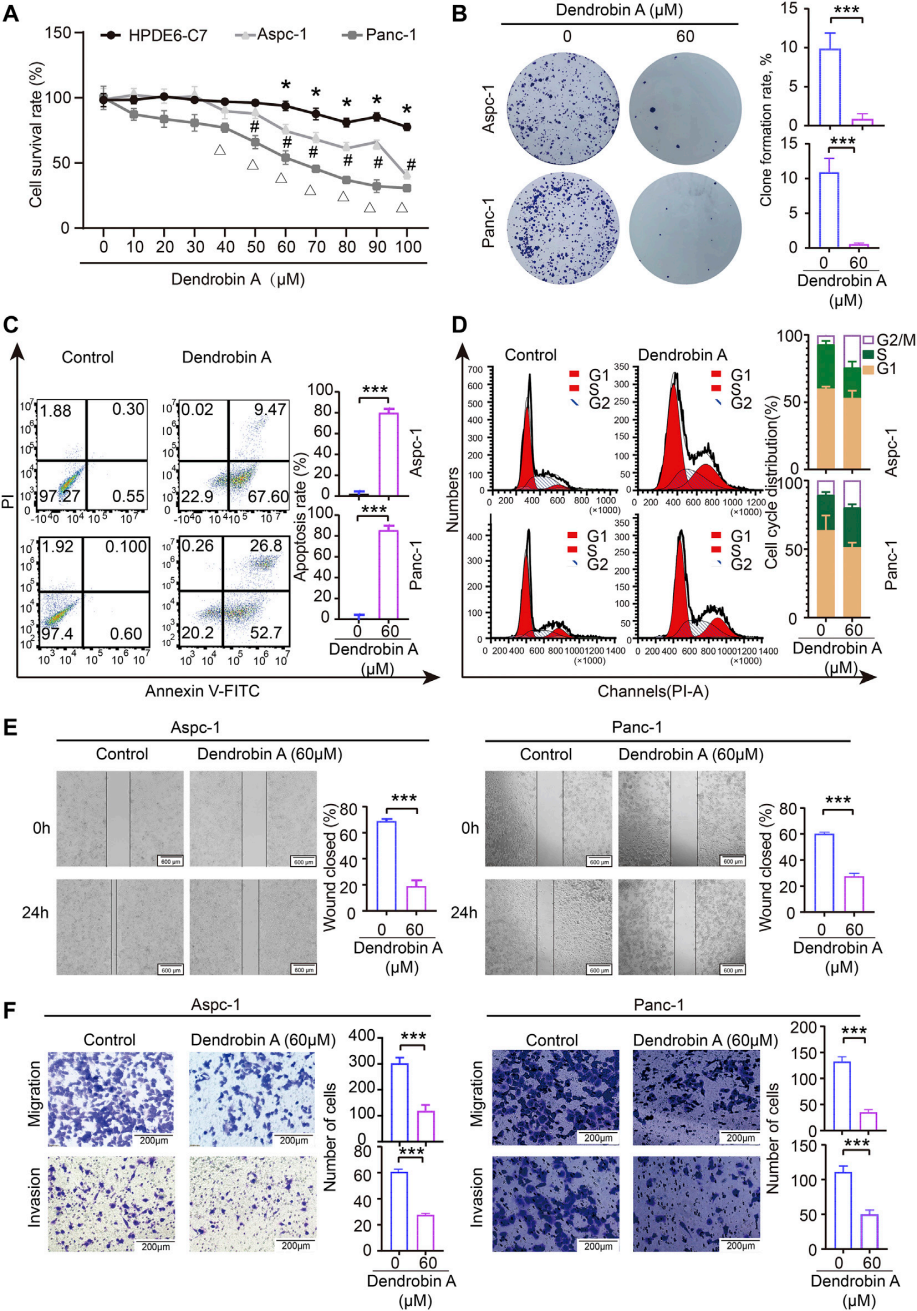
Effects of Dendrobin A on PDAC cell proliferation, cycle, apoptosis, migration, and invasion. **(A)** Results of CCK-8 assay; compared with 0 μM group, *p < 0.05, ^#^ *p* < 0.05, and ^Δ^ *p* < 0.05. **(B)** Results of plate clone formation assay, ***p < 0.001. **(C)** Results of apoptosis, ***p < 0.001. **(D)** Results of the cell cycle analysis. **(E)**. Results of cell migration; ***p < 0.001, bars = 600 μm. **(F)** Results of invasion; ***p < 0.001, bars = 200 μm.

Results of the CCK-8 assay indicated that Dendrobin A effectively inhibited the proliferation potentials of PDAC cells in a concentration-dependent manner but not for normal pancreatic cells (Figure 8A). Subsequent experiments were performed using Dendrobin A at 60 µM according to its IC 50 values. The colony formation assay results further confirmed the inhibitory effects of Dendrobin A on PDAC cells (Figure 8B). Considering that cell cycle and apoptosis are closely related to cell proliferation, we applied to flow cytometry to examine the effect of Dendrobin A on PDAC cell cycle and apoptosis. The results showed that the values of G_2_/M phases in PDAC cell lines Aspc-1 and Panc-1 have increased by 88.5% and 256% post-drug treatment, respectively (Figure 8C), and the total number of apoptotic cells in Dendrobin A-treated Aspc-1 and Panc-1 cells were increased to 77% and 79.5%, respectively (Figure 8D). We also tested the effects of Dendrobin A on PDAC cell migration and invasion. The wound healing and Transwell migration assay showed that Dendrobin A impaired PDAC cell migration and invasion abilities (Figures 8E, F).

## Discussion

TCM plays a special role in human life and health. Network pharmacology is an emerging technology to explore and identify the key targets of drugs, which provides an alternative way of exploring the application of TCM in the field of tumor treatment (Tang and Aittokallio, 2014; Asakawa et al., 2020). As one of the effective extracts of *D. nobile*, bibenzyl has various pharmacological activities such as anti-tumor, -inflammatory, and -diabetic (Zhao et al., 2018; Asakawa et al., 2020). Dendrobin A is a recently identified type of bibenzyl extracted from *D. nobile* (He et al., 2020), which has anti-cancer activities. However, its effects and mechanism of action on specified tumors remain unsolved. The goal of this work was to uncover the effects and mechanisms of Dendrobin A against PDAC.

Using SwissTargetPrediction and PharmMapper platform, 353 targets for Dendrobin A were identified. Subsequently, based on TCGA and GTXs databases, 3,131 DEGs between PDAC and normal pancreatic tissues were obtained. Finally, 90 potential targets of Dendrobin A against PDAC were identified. GO annotation indicated that these 90 genes were involved in metabolism-related biological processes and responses to drugs, while KEGG signals enrichment showed that cancer-related pathways and drug metabolism were enriched. To further explore the signatures of targets for Dendrobin A against PDAC, we overlapped these 90 genes with targets related to PDAC from the MalaCards database, and 13 key genes previously reported in PDAC were found, namely, *AURKA*, *CCNA2*, *ALB*, *LCN2*, *NQO1*, *CDA*, *PLAU*, *LGALS3*, *MMP2*, *REG1A*, *MMP7*, *MMP9*, and *PIK3CG*. Therefore, these 90 genes indicated that the role of Dendrobin A on PDAC is multifaceted.

Next, we established a PPI for these 90 genes using the STRING database. As a result, a close link among the PPIs was identified. We also found that a specific module consisted of 19 key genes, namely, *AURKA*, *KIF11*, *TK1*, *CDK1*, *CCNA1*, *AURKB*, *CCNA2*, *PLAU*, *SERPINA1*, *STSB*, *CTSS*, *REN*, *SELP*, *PPARG*, *LGALS3*, *MMP1*, *MMP9*, *NOS2*, and *LCN2*. The functional analysis further indicated that these 19 genes were indeed involved in pathways in cancer. Thus, these 19 genes provided a cue for Dendrobin A against PDAC.

Given that these 19 genes play a critical role in PDAC carcinogenesis, we analyzed the significance of these 19 genes in the OS of PDAC patients. However, eight genes displayed the OS-predicated values in PDAC. Among them, high expression of *AURKA*, *CCNA2*, *CDK1*, *KIF11*, *LGALS3*, *MMP1,* and *PLAU* is the risk factor, while high expression of *CCNA1* is a protective factor for PDAC. Indeed, *AURKA* (Gomes-Filho et al., 2020), *CCNA2* (Dong et al., 2019), *CDK1* (Piao et al., 2019), *KIF11* (Gu et al., 2022), *LGALS3* (Yi et al., 2020), *MMP1* (Chen et al., 2019), and *PLAU* (Kisling et al., 2021; Wang et al., 2022) are significantly associated with the prognosis of PDAC patients. Our current study provides focus targets of Dendrobin A against PDAC.

Cachexia, a typical characteristic of malignant tumors, especially for pancreatic cancer patients, is significantly associated with PDAC prognosis, treatment, and quality of life. Studies have reported the positive role of TCM in improving the cachexia status of tumor patients (Xu et al., 2021). In this study, we found a high association of Dendrobin A targets with PDAC cachexia. Among these genes, *PLAU* highlights the association with PDAC cachexia. Increasing reports showed that high expression of *PLAU* is a risk factor in various tumors, including PDAC (Ai et al., 2020; Fang et al., 2021; Li et al., 2021; Wang et al., 2022). Additionally, molecular docking found that Dendrobin A could be bound within the *PLAU* active pocket. In addition, after treating the PDAC cells with Dendrobin A, *PLAU* expression in PDAC cell lines was also dramatically changed. Thus, *PLAU* may have important molecular targeting value in anti-PDAC treatment. Indeed, an early report has proposed that *PLAU* is a potent target for the anti-PDAC using TCM (Zhao et al., 2020) and a potential prognostic marker in PDAC (Kisling et al., 2021; Wang et al., 2022). Since *PLAU* was one of the representative key targets identified in this study, the expression level of PLAU protein in PDAC and control paraneoplastic tissues in tissue microarrays was detected by immunohistochemistry. The results showed that the expression of PLAU was significantly higher in PDAC tissues, and its expression level correlated with the differentiation and lymph node metastasis status of PDAC. We also observed that PLAU protein levels were higher in female PDAC patients than in male PDAC patients, though the reason for this is unknown. Together, these results highlight the pathophysiological role of *PLAU* in PDAC.

Finally, we performed several *in vitro* experiments to test the feasibility of anti-PDAC using Dendrobin A. Similar to the expected results, we found that administration of PDAC cells with Dendrobin A led to substantially anti-cancer effects, as evidenced by the attenuated cell viability, migration, invasion, and enhanced apoptosis, and changed the cell cycle. Thus, the anti-PDAC properties of Dendrobin A are versatile.

In summary, our current study integrated network pharmacology, bioinformatics, and validation experiments, and found that Dendrobin A may exert its anti-PDAC action through multiple targets, and that *PLAU* may be a potential target for Dendrobin A against PDAC. Our study provides alternative evidence for the use of TCM to treat PDAC. However, a larger cohort and animal experiments are needed to verify these results.

## Data Availability

The original contributions presented in the study are included in the article/Supplementary Material, further inquiries can be directed to the corresponding authors.
