# Supplementary material for: Integrated analysis of Dendrobium nobile extract Dendrobin A against pancreatic ductal adenocarcinoma based on network pharmacology, bioinformatics, and validation experiments
Source: Front Pharmacol. 2023 Mar 1;14:1079539. doi: 10.3389/fphar.2023.1079539 (PMC10014786; doi:10.3389/fphar.2023.1079539)
Supplement: Supplementary file 1 [file DataSheet1.PDF]

Supplementary Material

Supplementary Figures

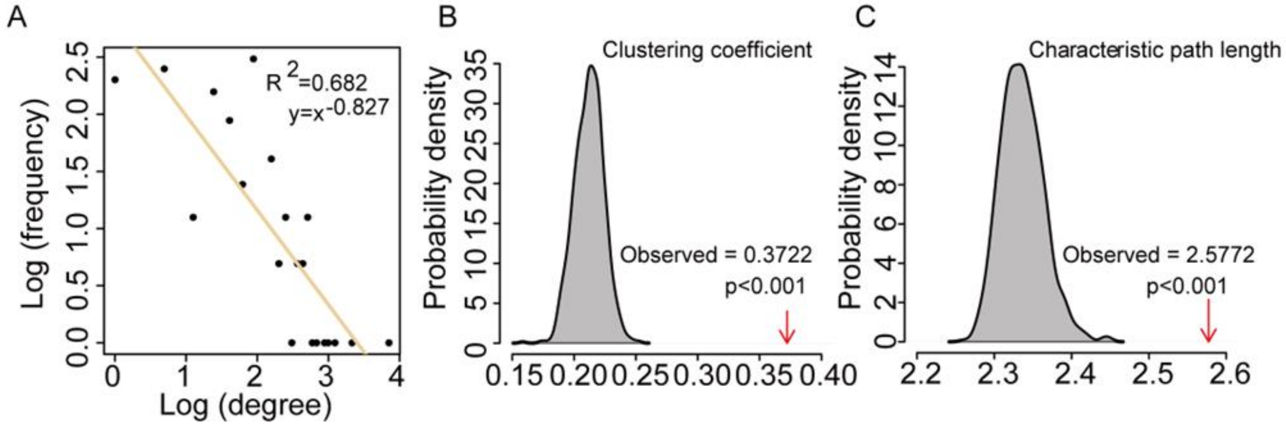

Supplementary Figure S1. PPI network profiles. (A) Connectivity distribution of PPI network nodes. (B) Network clustering coefficient compared with random network. (C) The characteristic path length of the network compared with that of the random network.

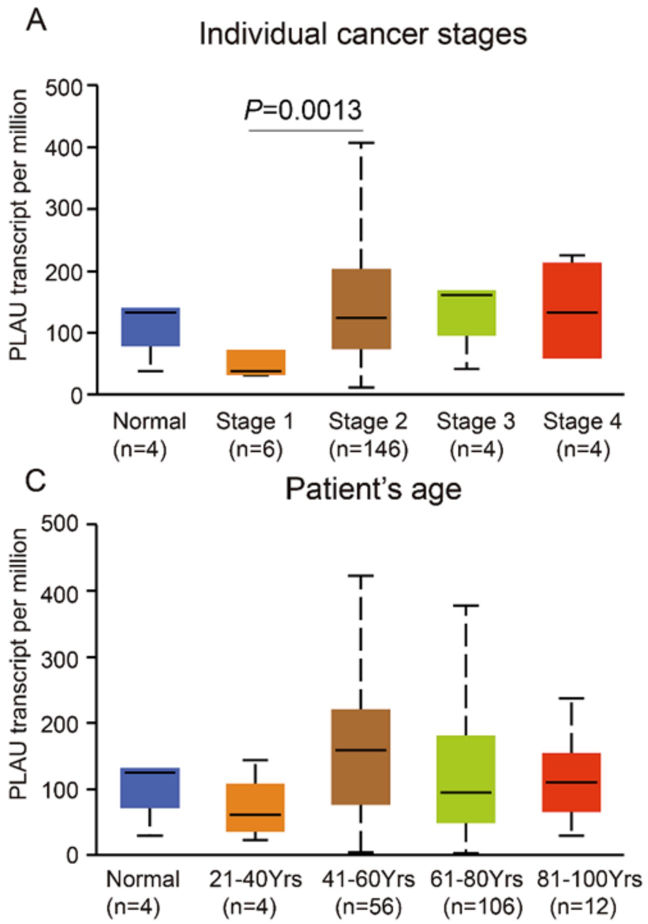

Supplementary Figure S2. The relation of *PLAU* expression to the clinicopathological features including individual cancer stage, nodal metastasis status, age and gender in pancreatic ductal adenocarcinoma patients.
